# Different pollinator assemblages ensure reproductive success of *Cleisostoma linearilobatum* (Orchidaceae) in fragmented holy hill forest and traditional tea garden

**DOI:** 10.1038/srep21435

**Published:** 2016-02-24

**Authors:** Xiang Zhou, Qiang Liu, Jessie Yc Han, JiangYun Gao

**Affiliations:** 1 Center for Integrative Conservation, Xishuangbanna Tropical Botanical Garden, Chinese Academy of Sciences, Mengla, Yunnan 666303, China; 2 University of Chinese Academy of Sciences, Beijing 100049, China

## Abstract

Orchids are generally recognized to have specialist pollination systems and low fruit set is often thought to be characteristic of the family. In this study, we investigated the reproductive ecology of *Cleisostoma linearilobatum*, an epiphytic tropical orchid, in a holy hill forest fragment and a traditional tea garden in SW China using comparable methods. *C. linearilobatum* is self-compatible and dependent on insects for pollination. Fruit production in natural conditions was both pollinator- and resource-limited. However, the natural fruit set remained stable over multiple years at both sites. Pollination observations showed that *C. linearilobatum* has a generalized pollination system and seven insect species were observed as legitimate pollinators. Although the visit frequencies of different pollinators were different in the two sites, the pollinator assemblages ensured reproductive success of *C. linearilobatum* in both study sites over multiple years. The results partly explain why *C. linearilobatum* is so successful in the area, and also suggest that holy hill forest fragments and traditional tea gardens in Xishuangbanna are important in preserving orchids, especially those with generalist pollination.

With more than 27,000 species and a wide distribution throughout all continents except Antarctica, orchids are one of the most successful groups of flowering plants on the earth (www.theplantlist.org). Both pollinators and mycorrhizal fungi are important to the diversification of orchids^1^,^2^, and pollinators in particular are considered to be important for speciation and colonization of new geographical areas^3^. Most orchids require pollinators for fruit set, and the fruit set of orchids is generally low, especially for epiphytic tropical species^4^,^5^,^6^. Orchids are generally recognized to have specialist pollination systems^7^,^8^, and pollination limitation has been the most frequently reported cause of reproductive failure^9^,^10^,^11^,^12^. Nevertheless, generalized pollination systems have been reported for a small number of orchid species and, in some case, they may provide stable reproductive success, particularly in habitats that are marginal for insect activity^13^,^14^.

Xishuangbanna is located on the northern margins of tropical Asia in southwestern China and has the largest area of tropical forest remaining in the country. It is in the Indo-Burma biodiversity hotspot and contains 16% of China’s vascular flora in less than 0.2% of the country’s total area (19,690 km2). The rapid expansion of monoculture crops in the last 20 years, particularly rubber, has resulted in serious forest fragmentation and threatens the exceptional biodiversity in this region^15^. Xishuangbanna is one of the most orchid-rich areas in China^16^,^17^. A total of 426 orchid species in 115 genera have been identified in the area, which amounts to 31% of all orchid species in China, and most species are threatened^18^. Beside the relatively large nature reserves, the ‘holy hills’ of the Dai ethnic group can also act as orchid refuges, especially for species over-collected elsewhere^18^. The major commercial crops are not significant habitats for orchids in Xishuangbanna, but traditional tea gardens, where tea is grown under a canopy of native trees, support a high diversity of orchids^19^,^20^.

In our systematic field surveys of orchid species diversity in Xishuangbanna, we found that *Cleisostoma linearilobatum* is the most abundant species in both ‘holy hills’ and traditional tea gardens at elevations between 1000–1600 m. We were curious about why this orchid was so successful in these different habitats and therefore decided to compare the reproductive ecology of *C. linearilobatum* in holy hills and traditional tea gardens. Here we present the results of our investigations, which addressed three principal questions: (1) Is the natural fruit set of *C. linearilobatum* different between holy hills and traditional tea gardens? Does its fruit production remain stable between years? (2) What are the pollinators of *C. linearilobatum*, and is the pollinator assemblage different between the two different habitats? (3) Why is *C. linearilobatum* so successful in this area?

## Results

### Floral phenology and morphology

**Figure 1:**
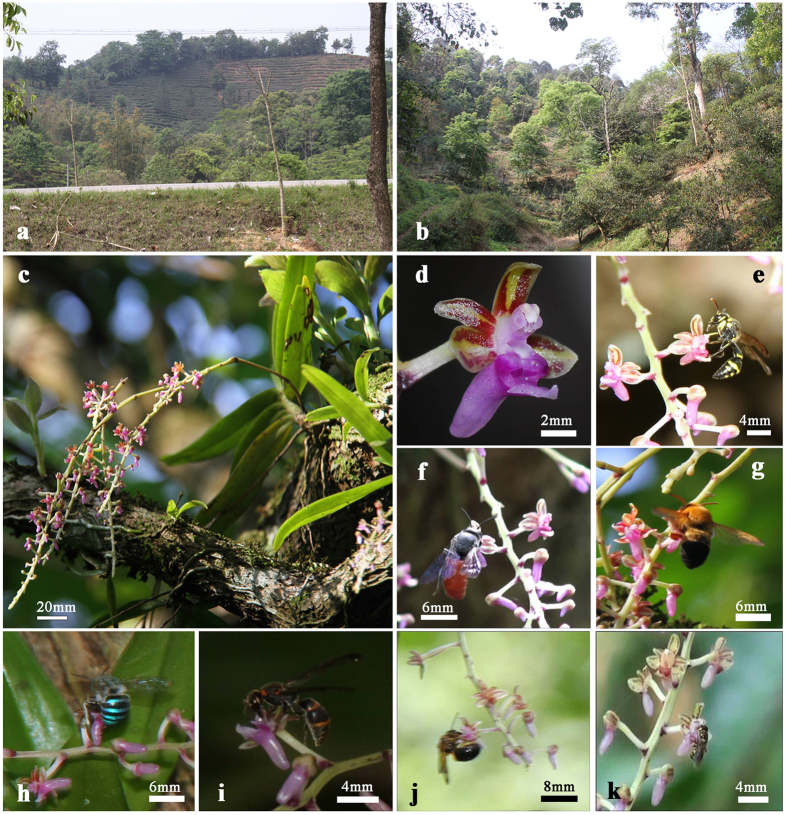
Two study sites, inflorescence, flower and different visitors of *Cleisostoma linearilobatum*. (**a**) The holy hill forest (HHF) is fragmented by modern tea monocultures; (**b**) The traditional tea garden (TTG) with many native shade trees; (**c**) Inflorescence; (**d**) Flower; (**e**) *Eumenes sp.*1; (**f**) *Prionyx sp*.; (**g**) *Megachile dimidiate*; (**h**) *Amegilla yunnanensis*; (**i**) *Vespa sp*.; (**j**) *Apis sp.*; (**k**) *Eumenes sp*. 2.

*Cleisostoma linearilobatum* was the most abundance orchid species in our study sites, with 66 mature individuals found in in HHF and 777 in TTG. It flowered from May to September (mainly early June to late July) in the study sites. It produces 1–3 paniculate pendulous inflorescences from the base of the stem. The panicle, with mean length of 17.72 ± 6.46 cm (*N* = 30), contains 57.00 ± 33.10 flowers (*N* = 30; Fig. 1c). Flowers were small, purplish-red in colour (Fig. 1d), and opened gradually from the bottom to the top of each panicle.

**Figure 2:**
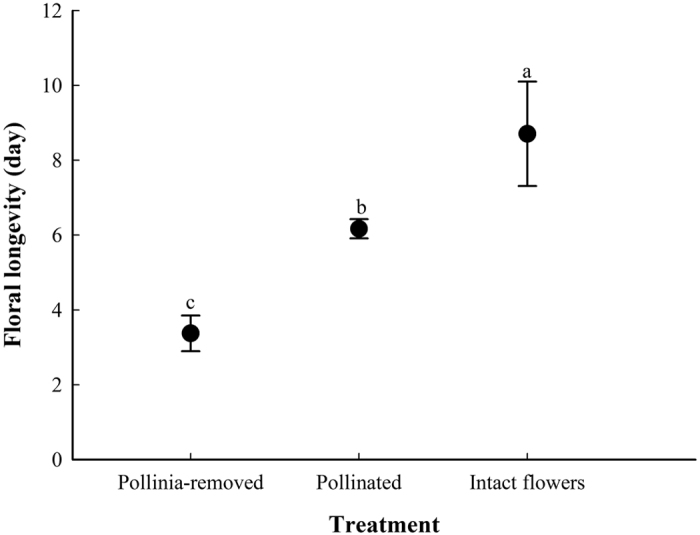
Floral longevity of *Cleisostoma linearilobatum* in different treatments. Statistically homogeneous groupings based on a one-way ANOVA are indicated by the same letter (**a–c**) above the bars.

Flowers of *C. linearilobatum* did not produce any odor detectable by a human nose. The spur was 4.02 ± 0.38 mm in length and 1.52 ± 0.16 mm in width (*N* = 60), longitudinally divided into two spaces by dissepiment, and filled up with 0.58 ± 0.37 μl of nectar with a sugar concentration of 36.23 ± 6.69% (*N* = 60). Flowers of *C. linearilobatum* normally lasted about 9 days (8.71 ± 1.39 d, *N* = 15), and floral longevity was shortened significantly both by pollinia-removal (3.38 ± 0.48 d, *N* = 15) and pollination (6.17 ± 0.26 d, *N* = 15), but pollinia-removal had more significant effects on floral longevity than pollination (P < 0.001; Fig. 2).

### Hand-pollination experiments and natural fruit sets

In the four hand-pollination treatments, no fruit was found in the bagging and emasculating treatments in either site (Table 1), suggesting that spontaneous autogamy and apomixis did not occur in *C. linearilobatum*. The fruit sets of the selfing and crossing treatments were not significantly different within or between HHF and TTG, but both were significantly higher than natural fruit sets in both study sites (*P*_*selfing & natural*_ < 0.001, *P*_*crossing & natural*_ < 0.001; Table 1). These results indicate that *C. linearilobatum* is completely self-compatible and dependent on insects for fertilization. The natural fruit sets of *C. linearilobatum* in the two study sites were stable over the 3 years from 2011 to 2013, with no significant differences between sites (F = 0.140, P > 0.05; Table 1) or among years (F = 0.138, P > 0.05; Table 1).

### Floral visitor observations

**Figure 3:**
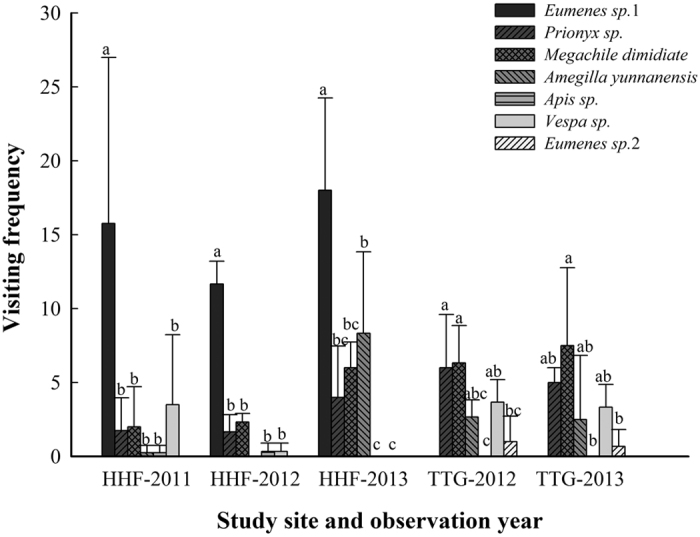
Mean visiting frequency of different pollinators to flowers of *Cleisostoma linearilobatum* in a holy hill forest (HHF) and a traditional tea garden (TTG) in different years. Statistically homogeneous groupings based on one-way ANOVA are indicated by the same letter (**a–c**).

Overall, 140 h of visitor observations were made at HHF over three years (2011 to 2013), and 84 h were made at TTG over two years (2012 and 2013). A total of seven insect species were identified as pollinators of *C. linearilobatum* in the two study sites. Among them, 6 species were observed in HHF and 5 species in TTG, with 4 species common to both sites. All 7 insects were observed visiting flowers for nectar and carrying pollinia away when leaving flowers. *Eumenes sp.*1, with the most visits observed, was the main pollinator of *C. linearilobatum* in HHF (Fig. 1e). It visited flowers more frequently and for longer periods than the other 5 species in three observation years, but was not observed in TTG (Fig. 3). In TTG, four insect species, *Prionyx sp.*, *Amegilla yunnanensis, Megachile dimidiate* and *Vespa sp.* (Fig. 1f–i) visited flowers of *C. linearilobatum* frequently and for longer periods, and there was no significant difference in their visiting frequencies between the two years (t_*Prionyx*_ = 0.577, P > 0.05, t_*Megachile*_ = −0.310, P > 0.05, t_*Amegilla*_ = 0.091, P > 0.05, t_*Vespa*_ = −0.378, P > 0.05; Fig. 3). Although these four insects were also observed in HHF, their visiting frequencies were significantly lower than in TTG, and the visiting frequency of *Amegilla sp*. was significantly different among years (F = 8.034, P < 0.05). A species of *Apis* (Fig. 2j) was occasionally observed visiting flowers of *C. linearilobatum* in HHF, but not in TTG; another species, *Eumenes sp*. 2 (Fig. 2k), was only observed in TTG. In 2013, diurnally bagged flowers showed no evidence of insect visitation at night, as their pollinia remained intact in the next morning.

## Discussion

Orchids are primarily pollination limited, and many factors may affect male and female reproductive success including floral traits^5^. *Cleisostoma linearilobatum* produces many flowers per inflorescence and these flowers have nectar as a reward for pollinators. The lack of fruit production in the bagging treatments indicated that *C. linearilobatum* was dependent on insects for pollination, and that spontaneous self-pollination did not occur. The fruit sets of selfing and crossing treatments were not significantly different, indicating that *C. linearilobatum* is completely self-compatible. Natural fruit sets were significantly lower than the fruit sets of any selfing and crossing treatments, which were also relatively low (54.08–67.00%, Table 1), suggesting that fruit production of *C. linearilobatum* in natural conditions was both pollinator- and resource-limited.

Most orchids have specialist pollination systems and about 60% have only one recorded pollinator^7^,^8^,^21^. The advantage of specialization is that pollen transfer between flowers is very efficient^22^,^23^, but these plants are often pollinator limited, which is the most frequent reported cause of reproductive failure in orchids^5^,^24^,^25^. Generalist pollination may alleviate this problem, partucularly in habitats that are marginal for insect activity^13^,^14^. Our observations suggested that *Cleisostoma linearilobatum* possess a generalized pollination system with 7 morphologically similar and effective pollinators in the two study sites. All 7 insects are very common in the study area and similar in body size. *Amegilla yunnanensis*, *Megachile dimidiate*, and many species of *Prionyx*, *Eumenes* and *Apis* are knew as common pollinators of other flowering plants. Although the pollinator assemblages and the visiting frequencies of different pollinators were different, the natural fruit sets of *C. linearilobatum* were stable over the years in both study sites (Fig. 3). This may partly explain why *C. linearilobatum* is so successful in this area.

Xishuangbanna is one of the orchid hotspots in China^16^,^17^. However, most species are threatened due to the habitat loss and over-collection. Although about two-thirds of orchid species occur in nature reserves, there are still 142 species recorded only outside the nature reserves, and most of these are found in forest fragments^18^. A recent investigation suggested that fragmentation had no significant impact on the species diversity and abundance of epiphytic orchids at elevations both above and below 1 000 m, but this may reflect the short history of forest fragmentation in this area^26^. Some studies have shown that fragmentation may directly affect pollinators of orchids and lead to reproductive failure in many orchid species^27^,^28^. Nevertheless, for *C. linearilobatum*, even in the seriously fragmented habitat of HHF, the natural fruit set remained stable over the years of the study and did not differ from TTG. Six insect species were observed as pollinators of *C. linearilobatum* in HHF. The visiting frequencies of different insects may differ between years, but all pollinators contributed to a stable fruit set.

In Xishuangbanna, the major commercial crops are rubber and tea. Rubber plantations are not suitable habitats for orchids in Xishuangbanna, but traditional tea gardens, where tea trees are grown under a canopy of native trees, support a high diversity of orchids^19^,^20^. About 5494 ha of these tea gardens still persist in Xishuangbanna at elevations of 1200–1800 m^29^ and they are also important for other native species^19^. This situation is very similar to the role of shade-coffee and cacao plantations in South America as refuges for tropical wild orchids^30^,^31^. Our study confirmed that in addition to the nature reserves, the ‘holy hills’ and traditional tea gardens in Xishuangbanna also act as orchid refuges, especially for those species with generalist pollination. As suggested^18^, continued protection with incentives for the continuation of the traditional conservation practice for holy hills and with traditional management for tea gardens are certainly needed and important.

## Methods

### Study sites

This study was conducted in a holy hill forest of the Dai minority and a traditional tea garden of the Jinuo minority in Xishuangbanna, Yunnan province, SW China. All holy hill forests and traditional tea gardens in Xishuangbanna are out of the nature reserves. Holy hills are sacred forested hills believed to be the dwelling place of the spirits of ancestors by Dai people, and are traditionally protected by customary rules. Nowadays, there are 328 holy hill forests remaining in the area, but they have been severely fragmented and vary greatly in size^20^. The traditional tea gardens, where tea is grown under a canopy of native trees, support a high diversity of orchids and other plants, and the premium paid for the tea they produce in a traditional manner has ensured that 5494 ha persist in Xishuangbanna at altitudes of 1200–1800 m^20^.

Our study site in holy hill forest (HHF) is located in Manwei village, Menghai county (21°59′ 41″N, 100°29′67″E; 1176 m alt.), a small fragment of tropical montane evergreen broad-leaved forest (1.73 hectare) with dominant trees including *Photinia serrulata*, *Pyrus betulifolia*, *Cinnamomum porrectum*, *Albizia odoratissima* and *Symplocos paniculata*, surrounded by newly established modern tea monocultures (Fig. 1a). The study site in a traditional tea garden (TTG) is located in Ya’nuo village, Jinghong city (21°59′46″N, 101°05′07″E; 1380 m alt.) and is contiguous with tropical montane evergreen broad-leaved forests (Fig. 1b). The remaining native shade trees in the tea garden include *Schima wallichii*, *Nephelium chryseum*, *Castanopsis echidnocarpa*, *Machilus gamblei*, *Alseodaphne andersonii*, *Meliosma arnottiana*, and *Macaranga indica*. Most of the tea trees, *Camellia sinensis* var. *assamica*, had been cultivated for more than 200 years and they have an average height of 2 m. The local people manage the tea garden in a very traditional way without using any pesticides, herbicide, and chemical fertilizer.

### Study species

*Cleisostoma* Blume is a genus with around 100 species distributed in the Asian tropics. Little is known about the pollination biology of *Cleisostoma* except that *C. parishii* was said to be autogamous^32^. *Cleisostoma linearilobatum* is a small epiphytic orchid widely distributed in Southeast Asia including Xishaungbanna in Southwest China^33^. In our study areas, it usually grows on tree trunks in tropical montane evergreen broad-leaved forests at altitudes of 1000–1600 m.

In our study sites, a total of 13 orchid species in 10 genera were found in HHF, while 38 orchids of 17 genera were found in TTG, growing both on tea trees and shade trees^20^. The main study period was during the flowering and fruiting seasons of *C. linearilobatum* from 2011 to 2013.

### Floral phenology and morphology

Phenology was initially monitored monthly in HHF from May 2011 to August 2012, then flowering phenology was observed daily in both HHF and TTG during the flowering seasons of 2011 and 2012. The floral longevity of pollinia-removed, pollinated and intact flowers was compared by marking 15 flowers from 5 individuals for each treatment. The floral morphology was studied in HHF in 2011. The inflorescence numbers of 30 randomly selected individuals and flower numbers of 30 randomly selected inflorescences were counted. The flower size and sizes of separate parts including gynostemium, dorsal and lateral sepals, petal, labellum and spur, were measured for 30 randomly selected flowers using a vernier caliper. Nineteen randomly selected inflorescences were bagged before anthesis to study the nectar secretion. We used 10-μL Sigma “micro-cap” calibrated capillary tubes (Sigma Chemical Co., St. Louis, USA) to measure the nectar volumes of 60 newly opened flowers during 13:00–14:00 on 5th June, 2011, and the nectar sucrose concentration of each flower was measured with a hand-held, temperature-compensated refractometer (Eclipse, Bellingham & Stanley Ltd., UK) at the same time.

### Hand-pollination experiments and natural fruit sets

The breeding system of *C. linearilobatum* was evaluated by different hand-pollination treatments in the two study sites in 2011. The treatments were: (i) bagging, inflorescences were bagged throughout without pollination; (ii) emasculating, pollinia were removed before flowers opened and then inflorescences were bagged throughout; (iii) selfing, inflorescences were bagged before flower opening and flowers were hand-pollinated with pollinia from the same flower, and then inflorescences were bagged again; (iv) crossing, inflorescences were bagged before flower opening and flowers were hand-pollinated with pollinia of another individual, and then inflorescences were bagged again.

All treatments were conducted in HHF and TTG during 7–13 and 9–15 June 2011, respectively. The fruit set of each treatment was counted about 4 weeks later (28–30 July). The natural fruit sets of *C. linearilobatum* in the two study sites were investigated by randomly marking inflorescences from different individuals for three consecutive years (2011–2013). The numbers of flowers, inflorescences and individuals that were used in hand-pollination treatments and investigations of natural fruit set are summarized in Table 1.

### Floral visitor observations

Floral visitor observations were made during the flowering seasons for three consecutive years (2011–2013) in HHF and two years in TTG (2012–2013). Observations were made from 6:00 am to 20:00 pm each day. Five inflorescences of different individuals were randomly selected for each observation. The behaviors of the flower visitors, time and frequency of visitation, number of flowers visited per visitation, numbers of flowers visited per inflorescence, and time length visiting a single flower, were observed and recorded. All visitor species were photographed and captured for identification. To exclude the possibility of nocturnal pollinators of *C. linearilobatum*, additional observations were made on five inflorescences randomly selected each in HHF and TTG during the flowering season in 2013. The inflorescences were bagged diurnally from 6:00 am to 20:00 pm, and kept open nocturnally. All flowers were monitored twice at 6:00 am and 20:00 pm to check if removal and deposition of pollinia had occurred.

### Statistical data analysis

The difference in floral longevity among treatments and in visiting frequency in HHF among three consecutive years was analyzed using one-way ANOVA, and the visiting frequency in TTG between 2012 and 2013 was compared using a Paired-Samples T-test. A General Linear Model was used to explore the relationships between natural fruit set among different years and between study sites, and the fruit set of different hand-pollination treatments, and between study sites. All statistical analyses were performed by SPSS ver. 13.0 for Windows.

## Additional Information

**How to cite this article**: Zhou, X. *et al.* Different pollinator assemblages ensure reproductive success of *Cleisostoma linearilobatum* (Orchidaceae) in fragmented holy hill forest and traditional tea garden. *Sci. Rep.* **6**, 21435; doi: 10.1038/srep21435 (2016).

## Figures and Tables

**Table 1:** Natural fruit sets of *Cleisostoma linearilobatum* in a holy hill forest (HHF) and a traditional tea garden (TTG) over 3 years from 2011 to 2013, and the fruit sets of different hand-pollination treatments in 2011 (mean±SD).

Study sites	Natural fruit set			Fruit set of hand-pollination treatments in 2011			
	2011	2012	2013	Bagging	Emasculating	Selfing	Crossing
HHF							
Fruit set (%)	8.48 ± 4.84^b^	10.24 ± 5.24^b^	9.06 ± 7.11^b^	0^c^	0^c^	58.45 ± 31.27^a^	54.08 ± 32.61^a^
Inflorescence/plants (flowers)	30/30 (1417)	24/24 (890)	30/30 (922)	10/10 (568)	10/10 (80)	14/14 (75)	10/10 (56)
TTG							
Fruit set (%)	8.42 ± 6.79^b^	8.48 ± 6.79^b^	10.31 ± 7.70^b^	0^c^	0^c^	65.00 ± 14.34^a^	67.00 ± 21.63^a^
Inflorescence/plants (flowers)	31/31 (1934)	23/21 (1042)	31/31 (1726)	10/10 (448)	10/10 (90)	10/10 (100)	10/10 (100)

Statistically homogeneous groupings based on General Linear Model analysis are marked by the same letter (a–c).
